# Rapid Treatment With Veno-Venous Extracorporeal Membrane Oxygenation in a Patient Who Developed Acute Respiratory Distress Syndrome After Drowning

**DOI:** 10.7759/cureus.102849

**Published:** 2026-02-02

**Authors:** Alison Thornton, Yatin Srinivash Ramesh Babu, Alessandra Ottley, Ramon Gil, Faraz Jaffer

**Affiliations:** 1 Medicine, Nova Southeastern University Dr. Kiran C. Patel College Of Osteopathic Medicine, Fort Lauderdale, USA; 2 Surgery, HCA Florida Westside Hospital, Plantation, USA; 3 Critical Care, HCA Florida Westside Hospital, Plantation, USA

**Keywords:** acute respiratory distress syndrome [ards], drowning, ecmo, intensive care, vv ecmo

## Abstract

This is a case of a 76-year-old male with a past medical history of hypertension, chronic kidney disease (CKD), transient ischemic attack (TIA) status post tissue plasminogen activator (tPA), bilateral carotid stenosis, and seizure disorder who was brought into a South Florida emergency department by emergency medical services after a drowning incident secondary to a seizure. It was found that he had low oxygen saturation, a low Glasgow coma score (GCS), and was intubated and mechanically ventilated. CT of the chest and arterial blood gas (ABG) determined that he had acute respiratory distress syndrome (ARDS) with severe hypoxemia despite mechanical ventilation. The patient was started on venovenous extracorporeal membrane oxygenation (V-V ECMO) within hours of being brought in by EMS and was successfully weaned from ECMO on day three. The patient eventually tolerated room air by day seven. This report highlights the importance of early initiation of ECMO in cases of ARDS. More studies are needed to determine if using ECMO after drowning can be used more commonly in practice, as in infectious cases.

## Introduction

Acute respiratory distress syndrome (ARDS) is a life-threatening ailment characterized by severe hypoxemia, diffuse pulmonary infiltrates, and reduced lung compliance, requiring venovenous extracorporeal membrane oxygenation (V-V ECMO) as a rescue therapy when inadequate oxygenation cannot be maintained by conventional mechanical ventilation. Early initiation of ECMO has been shown in various studies to bolster the efficacy of lung-protective ventilation strategies and improve overall outcomes in rapidly deteriorating patients [1-4].

ECMO can be administered in different modes. Patients with isolated respiratory failure would be appropriate candidates for V-V ECMO; on the other hand, veno-arterial (VA) ECMO is utilized when cardiac support is also necessary for the patient. Theoretically, in situations of combined respiratory and cardiac compromise, comprehensive hemodynamic and oxygenation support may be provided by VA-ECMO, thus making careful patient selection paramount to optimize clinical outcomes.

The clinical course for V-V ECMO patients with ARDS typically ranges from 7-10 days, allowing the lung to recover while the ventilator maintains lung-protective ventilation. Thus, gradual weaning from support is undertaken as ABG parameters and clinical stability continue to improve over time [2]. Settings for V-V ECMO include adjusting blood flow rate, sweep gas flow, and the fraction of inspired oxygen (FiO₂) delivered via the oxygenator. Clinicians usually set flow rates at 4-6 L/min and change the parameters through serial arterial blood gas (ABG) measurements to augment an individual's oxygenation and carbon dioxide removal [5].

Infectious etiologies are primarily implicated in the development of ARDS. Inflammatory responses due to viral organisms such as COVID-19 and influenza, as well as bacterial agents such as Mycoplasma pneumoniae and tuberculosis, along with aspiration-related events, frequently activate the syndrome [1,4,6]. The classic clinical presentation arises with acute onset of dyspnea and hypoxemia, which is resistant to supplemental oxygen, with measured respiratory compliance often falling below 40 mL/cmH₂O [2,4,6]. Moreover, patients may have complications secondary to the ARDS itself, including but not limited to multiorgan failure secondary to sepsis or complications related to mechanical ventilation and ECMO administration, such as barotrauma and coagulopathy [2,7].

In the case of drowning, water aspiration directly triggers ARDS. It washes out pulmonary surfactant, damages the alveolar-capillary membrane, triggers pulmonary edema, and releases inflammatory mediators. Gas exchange is damaged as pulmonary surfactant gets diluted in freshwater or when seawater creates osmotic gradients, which results in ARDS-like syndrome for up to one-third of the survivors [8].

Diagnostic tools such as the PREdicting dEath for SEvere ARDS on V-V ECMO (PRESERVE) mortality risk score aid in patient stratification, as it incorporates factors such as immunocompromised status and duration of pre-ECMO ventilation [9].

In this report, we discuss a case of a 76-year-old male with a past medical history of hypertension, chronic kidney disease, and seizure disorder who, after a drowning episode, presented with severe, refractory ARDS necessitating initiation of V-V ECMO. His clinical management has illustrated the importance of early ECMO implementation and the associated hurdles and possible advantages pertaining to ECMO use in complex clinical scenarios.

## Case presentation

A 76-year-old male with a past medical history of hypertension, chronic kidney disease (CKD), transient ischemic attack (TIA) status post tissue plasminogen activator (tPA), bilateral carotid stenosis, and seizure disorder was brought into a South Florida emergency department by emergency medical services after a drowning incident secondary to a seizure. The patient was found in a pool after having an apparent seizure. The incident was not witnessed, but the approximate exposure to water during the seizure was 2 minutes. He was found by a neighbor to be minimally responsive in the pool. The patient's last known seizure was six months before the episode, and the patient was compliant with all anti-epileptic medications: levitracetum and lacosamide. Rescue services arrived, where his oxygen saturation was 60% on room air, and improved to 80% on oxygen. On arrival to the emergency department (ED), his vitals were oxygen saturation: 74% on a 15 L nonrebreather mask, pulse: 98 bpm, respiratory rate: 26 bpm, BP 172/95 mmHg, T: 35.7 °C. He never lost a pulse and had multiple bouts of vomiting. He was moving all extremities, could follow commands, but was severely hypoxic with SpO_2 _in the 50-70s; thus, he was intubated.

Investigations

**Figure 1:**
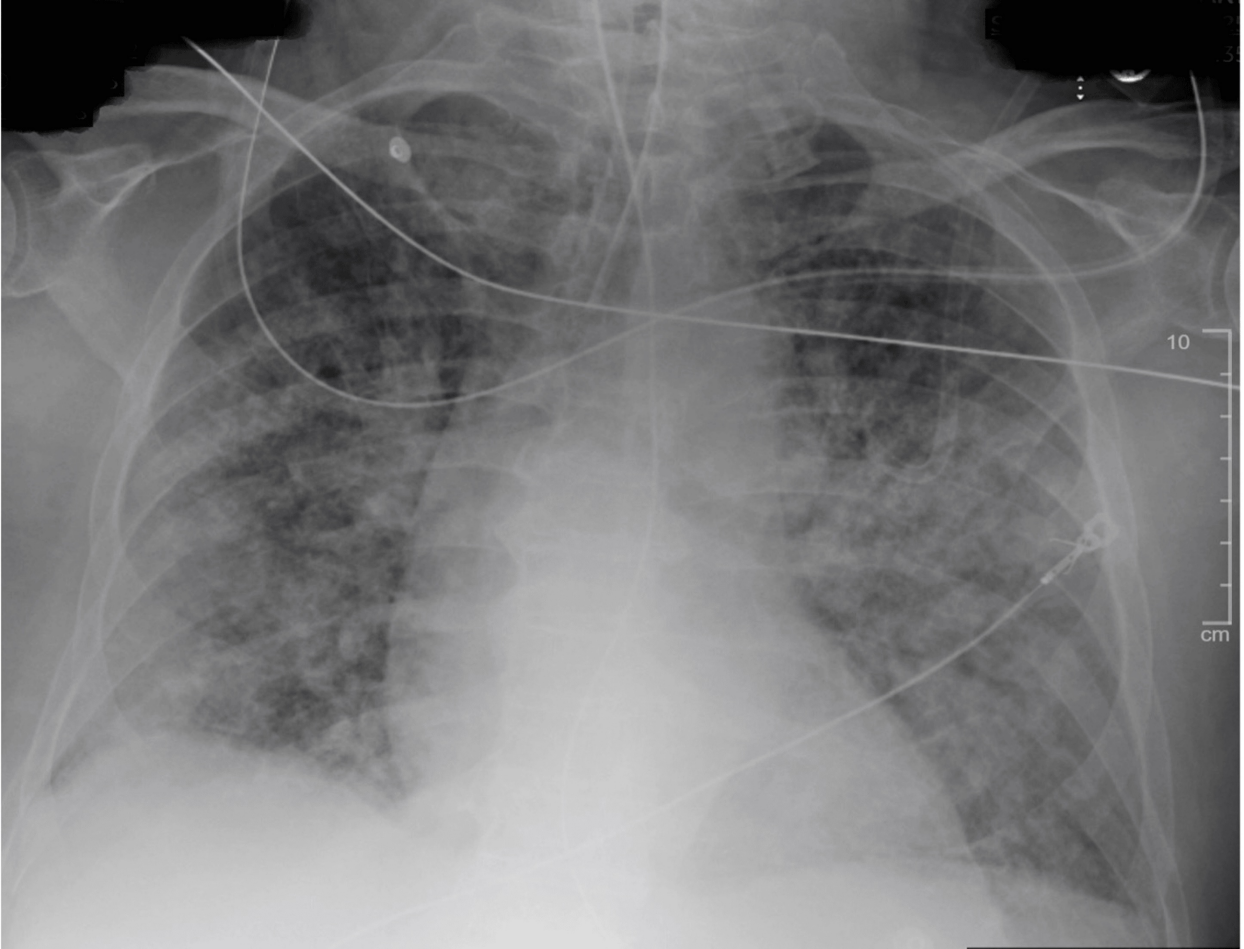
Chest X-ray on arrival to the emergency department. Patchy opacities are seen bilaterally.

**Figure 2:**
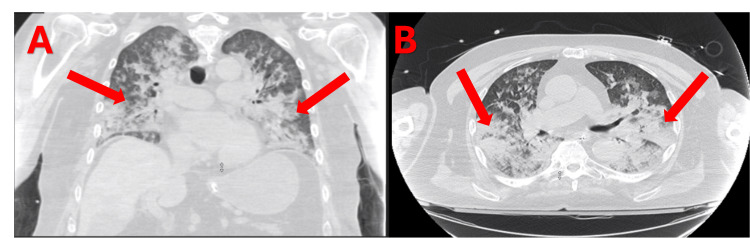
CT chest depicting patchy opacities bilaterally in coronal (A) and transverse (B) views.

In the emergency department, he met sepsis criteria, due to his vitals and a white blood cell count of 13.3 × 10^3^/uL (Table 1), and received lactic acid level testing, blood cultures, an intravenous fluid bolus, and antibiotics. An electrocardiogram displayed sinus tachycardia with 1st-degree AV block, a heart rate of 112, with no ST elevations, ST depressions, or QTc prolongations. A chest x-ray and CT of the chest revealed diffuse patchy opacities bilaterally (Figures 1, 2). The patient was found to have ARDS with severe hypoxemia despite mechanical ventilation. The patient remained hypoxic despite trials of paralysis, inverse ratio ventilation settings, and high-PEEP ARDS targeted ventilator settings. A STAT consult was made to cardiothoracic surgery (CTS) and critical care. The arterial blood gas drawn at this time can be seen in Table 2, with a trend throughout the patient’s stay. The patient was immediately brought to the operating room for V-V ECMO.

**Table 1:** White blood cell count trend during hospital stay Reference range: 4.5 to 11x10^3^/uL L: Low, below the lower limit of the reference range; H: High, Above the upper limit of the reference range

Day	WBC (10³/uL) (Reference range: 4,500-11,000 µL)
Day 1	13.3 H
Day 1	6.8
Day 2	8.5
Day 2	11.4 H
Day 2	15.2 H
Day 2	15.8 H
Day 2	15.4 H
Day 2	14.8 H
Day 3	13.7 H
Day 4	11.5 H
Day 5	12.2 H
Day 6	9
Day 7	6.9
Day 8	8.1
Day 9	9.8

**Table 2:** Arterial blood gas trend throughout hospital stay. pH reference range: 7.35-7.46 PaO₂/FiO₂ ratio reference range: ≥400 L: Below the lower limit of the reference range; H: Above the upper limit of the reference range; V-V ECMO: Venovenous extracorporeal membrane oxygenation; ARDS: Acute respiratory distress syndrome

Date	Time	pH (reference range: 7.35-7.46)	PaO₂/FiO₂ Ratio (reference range: ≥400)	Notes
Day 1	16:24	7.21 L	46.5 L	Critical pH and P/F
Day 1	20:32	7.32 L	387.6	—
Day 2	0:34	7.33 L	344.3	—
Day 2	4:18	7.38	213.3	—
Day 2	4:22	7.28 L	152.9 L	Low pH, Moderate P/F
Day 2	4:24	7.29 L	2053.8 H	Low pH
Day 2	10:22	7.35	274.3	—
Day 2	14:44	7.41	353	—
Day 2	20:18	7.38	207.8	—
Day 3	0:00	7.34 L	197.2 L	Borderline values
Day 3	4:00	7.34 L	219.4	Low pH
Day 3	8:20	7.37	204.8	—
Day 3	11:58	7.38	218.3	V-V-ECMO decannulated after this ABG
Day 3	17:15	7.38	169.8 L	Moderate ARDS
Day 3	21:50	7.45	165.3 L	Moderate ARDS
Day 4	14:46	7.46 H	126.0 L	Low P/F
Day 5	6:01	7.45	125.9 L	Low P/F
Day 5	12:48	7.46 H	205.9	—

Treatment 

CTS took the patient to the operating room and placed an Avalon 32 French cannula by accessing the right internal jugular for V-V ECMO catheter. The patient was started with a flow of 4 L per minute. There was immediate improvement in the patient's oxygenation and ventilation. Transesophageal echocardiography (TEE) estimated an ejection fraction of 45-50% with anteroseptal and basal inferoseptal hypokinesis. The patient was placed on low tidal volume, low respiratory rate, and high positive end-expiratory pressure ventilator settings to rest the lung and was admitted to the cardiovascular intensive care unit (CVICU). 

On day two of hospitalization, the ECMO settings were flow: 3.2L, sweep: 2, FiO_2_: 40%. The patient was being weaned off as tolerated. The patient’s white count was 15.4, with concern for pneumonitis. The vancomycin started the day before was discontinued due to nephrotoxic effects, and he was continued on piperacillin/tazobactam. The chest x-ray was mostly unchanged from the previous day with multifocal patchy opacities in the bilateral lungs, concerning for multifocal pneumonia and/or pulmonary edema. No pleural effusion was seen.

**Figure 3:**
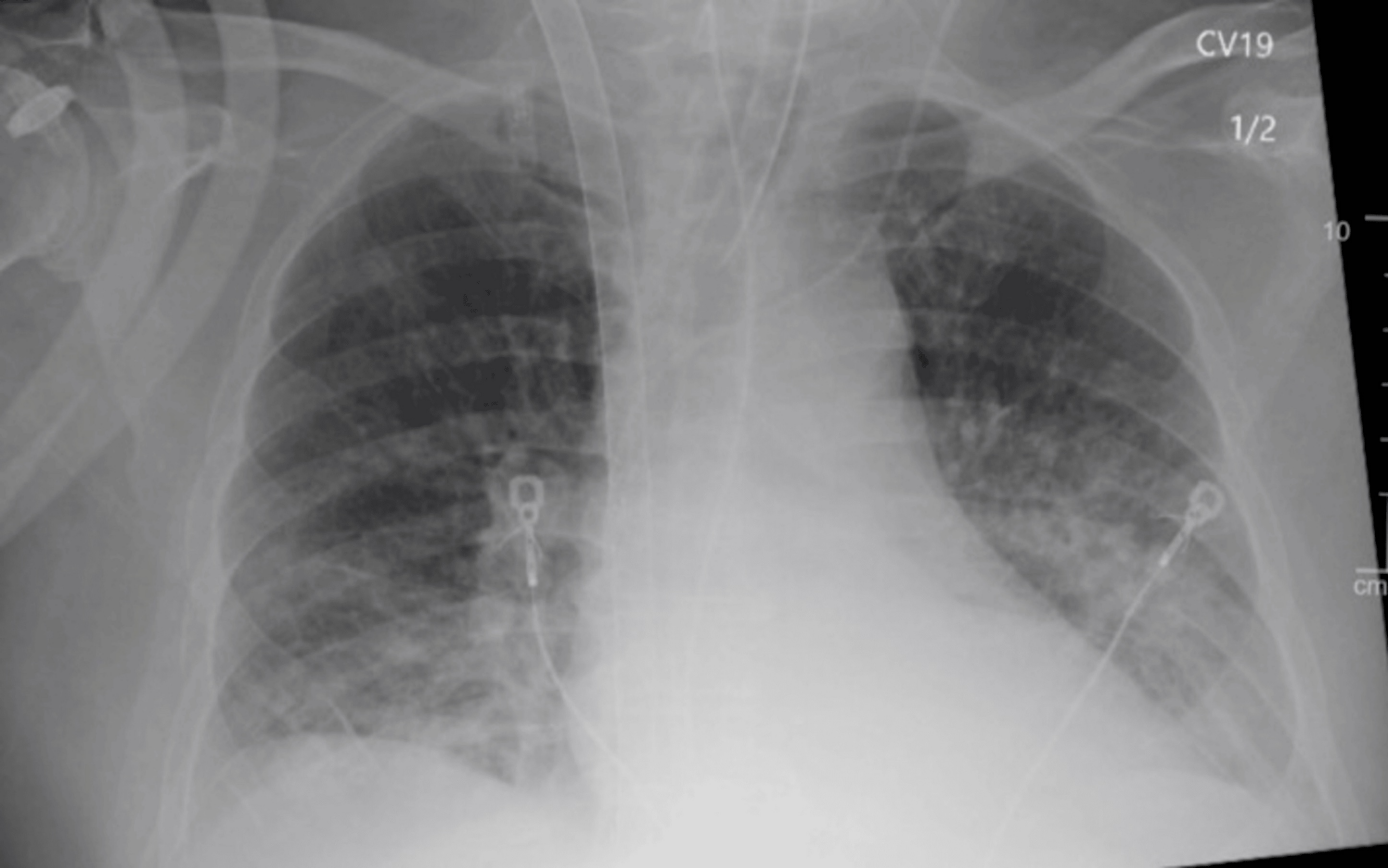
Chest X-ray just prior to V-V ECMO decannulation. Improved aeration is seen bilaterally.

On day three, the ECMO settings were: flow: 2L, sweep: 0, and FiO_2_: 0%. The arterial blood gas pH normalized from 7.21 on day 1 to 7.45, and the pO_2_/FiO_2_ ratio increased from 46 on day 1 to 218, as seen in Table 2. The chest x-ray remained unchanged from the previous day. The patient tolerated weaning, and mechanical circulatory support was decreased slowly to 0. Both lines were clamped, and the patient was decannulated successfully. A chest x-ray taken just prior to decannulation is shown in Figure 3. The patient was on minimal ventilation settings and was eventually extubated. However, he was still on norepinephrine and vasopressin to maintain a mean arterial pressure over 65. There were no V-V ECMO-related complications during cannulation, such as bleeding, thrombosis, or infection.

By day four of hospitalization, the patient was placed on a high-flow nasal cannula as tolerated. He was alert but agitated. The patient had no neurological deficits and was following all commands on physical exam, despite the drowning incident. The arterial line and central venous catheter were removed. Lactic acidosis resolved, and the white count was trending down. Chest x-ray continued to show bilateral lung patchy opacities likely from edema with low lung volumes and small pleural effusions.

On days 5-7, he continued to improve, with only one day requiring bilevel positive airway pressure (BiPAP), but mostly on a nasal cannula for oxygenation and eventually tolerating room air. He completed his antibiotic course with the white count normalizing and negative blood and sputum cultures. The trend in the patient's white blood cell count is shown in Table 1. 

On day eight, the patient no longer required intensive level care. He was alert and oriented, mentating well, and could recall the events from his hospital stay. On day 10, he was discharged to a skilled nursing facility for rehabilitation. 

## Discussion

This report explores the case of a 76-year-old male who presented to the emergency department after a drowning incident secondary to a seizure. This patient required mechanical ventilation due to low oxygen saturation and a low Glasgow Coma Scale (GCS). The chest x-ray and CT of the chest showed diffuse patchy opacities bilaterally, and they were diagnosed with ARDS with severe hypoxemia despite mechanical ventilation. It was determined that the patient would be started on V-V ECMO.

Recent data suggest that the timing of ECMO initiation strongly correlates with survival, especially in patients with severe ARDS, particularly those cannulated within the first five days of invasive mechanical ventilation, demonstrating significantly lower 28-day and in-hospital mortality rates than those with delayed initiation [2,10]. Early ECMO enabled ultra-protective ventilation strategies, thereby reducing barotrauma and volutrauma to the compromised lung parenchyma [11]. While many of the patients in these studies had COVID-19, some new studies are exploring its use in drowning victims. This strategy can be observed in a case report of an 18-year-old drowned victim who was successfully resuscitated using pre-hospital VA-ECMO. The patient underwent rapid cannulation while in the field after 24 minutes underwater and was then transported to a nearby intensive care unit for further management. Spending a total of five days of cannulation time, the young patient had favorable neurological outcomes after weeks of rehabilitation. This case underscores the crucial importance of timely cannulation and even prehospital ECMO in drowning cases within a well-equipped emergency response system [12]. Another case was reported of a 66-year-old female with cardiopulmonary arrest and drowning, who was started on ECMO two days after resuscitation due to continued severe respiratory failure from ARDS. She was on ECMO for 10 days. Our patient had a similar story; however, they were initiated on V-V ECMO within hours of arrival to the ED. Our patient was also in severe ARDS but was able to be weaned off V-V ECMO within two days of initiation [13].

The early initiation of V-V ECMO in this patient was likely a factor in his quick recovery. In a systematic review focusing on the mortality rates in patients with ARDS on ECMO therapy, it was seen that patients were on ECMO for an average duration of 8-16 days. Also, the duration of mechanical ventilation prior to ECMO had a median ranging from 1 to 8 days. Our patient was initiated on ECMO within hours of arrival to the hospital and was able to be weaned off ECMO within three days. However, most patients in these studies were on ECMO due to infectious causes of ARDS as opposed to other causes, like in our patient [3].

The pathophysiology of drowning-induced ARDS differs from more common infectious or inflammatory causes. Aspiration of water leads to immediate surfactant washout, disruption of alveolar-capillary integrity, and the development of non-cardiogenic pulmonary edema. Freshwater aspiration dilutes surfactant and causes alveolar collapse, while saltwater aspiration creates significant hyperosmolar gradients that draw fluid into the alveolar space, contributing heavily to the patient’s ventilation-perfusion mismatch. This insidious form of ARDS, when compared to infectious/inflammatory etiologies, pushes for earlier consideration of V-V ECMO, which in turn provides a means of rapid oxygenation and manual carbon dioxide clearance while allowing the lung tissue to rest and heal under ultra-protective ventilation settings [8]. However, one study found that ECMO in drowning patients did not change mortality significantly [14]. More studies are needed with this specific cause of ARDS and the use of V-V ECMO to determine how useful it can be in common practice.

The PRESERVE score can help intensive care physicians select candidates for ECMO with severe ARDS and determine survival probability. Higher scores indicated a higher probability of death. Components of the PRESERVE score include age, BMI, immunocompromised, sequential organ failure assessment (SOFA) >12, mechanical ventilation greater than 6 days, no prone positioning before ECMO, positive end expiratory pressure (PEEP) <10 cm H_2_O, and plateau pressure >30 cm H_2_O [9].

Common complications of ECMO are vascular and hematological related, such as bleeding, thrombosis, thrombocytopenia, and hemolysis. Making sure that patients are balanced appropriately with anticoagulation can help prevent these complications [15].

Limitations

The limitations of our case report involve the fact that there are limited studies to compare our patient to. While there are many studies about ECMO following cases of infectious ARDS, there are limited studies about ECMO for the treatment of ARDS due to drowning. More research is needed to determine if this method of early initiation of ECMO in drowning victims with ARDS can be used in common practice.

## Conclusions

This case of a drowning patient secondary to a seizure highlights the importance of early initiation of ECMO for faster recovery from ARDS. PRESERVE scores can help physicians determine the survival probability among patients with severe ARDS. Risks and benefits must be weighed before initiating patients on ECMO. As of now, there are limited studies on the use of ECMO after drowning, especially in older patients. Larger studies exist for pediatric populations. The early initiation of ECMO in this patient is likely one of the main reasons he recovered as quickly as he did. Further studies are needed to determine if this method can be used more commonly as an earlier treatment method for improved outcomes.
